# A weighted denoising method based on Bregman iterative regularization and gradient projection algorithms

**DOI:** 10.1186/s13660-017-1551-4

**Published:** 2017-11-09

**Authors:** Beilei Tong

**Affiliations:** 1 School of Mathematical Sciences, University of Science and Technology of China, Hefei, Anhui 230026 P.R. China; 2 School of Science, Southwest University of Science and Technology, Mianyang, Sichuan 621010 P.R. China

**Keywords:** 65F22, 68U10, 35A15, 65K10, 52A41, total variation, optimization, image denoising, Bregman distance, gradient projection method

## Abstract

A weighted Bregman-Gradient Projection denoising method, based on the Bregman iterative regularization (BIR) method and Chambolle’s Gradient Projection method (or dual denoising method) is established. Some applications to image denoising on a 1-dimensional curve, 2-dimensional gray image and 3-dimensional color image are presented.

Compared with the main results of the literatures, the present numerical results of the proposed method are improved.

## Introduction

In this paper, we consider the image denoising problems. The objective is to find the unknown true image $u \in R^{n}$ from an observed image $g \in R^{n}$ formed as the follows:

1.1 $$ g = u + n, $$

where $n \in R^{n}$ refers to the additive white gaussian noise. To remove the additive white gaussian noise well, Rudin, Osher and Fatemi (ROF) first proposed the total-variation (TV) regularization denoising model in [1]. This denoising model is actually the optimization of the ROF functional:

1.2 $$ \min_{u} \Vert \nabla u \Vert _{1} + \frac{1}{2\lambda} \Vert u - g \Vert _{2}^{2}, $$

where, for the continuous case, *i.e*. $u \in L^{1}(\Omega )$, Ω is an open subset of $R^{n}$. Here, ${\Vert \cdot \Vert _{1}}$ denotes the $L_{1}$ norm. We also denote $\Vert \nabla u \Vert _{1}$ by $\operatorname{TV}(u)$. The TV regularization model had been popular from then on for it can preserve the edges and the details as denoising [2, 3].

There are various excellent algorithms to solve the ROF denoising model [4–9]. In this paper, we consider two state-of-the-art denoising methods, *i.e*. Chambolle’s gradient projection denoising algorithm [4] and Osher *et al*.’s Bregman iterative regularization method [5]. Chambolle solved the ROF model in the dual field. The Bregman iterative regularization method in [5] gave a significant improvement over standard ROF models by taking back useful information to the denoising results. Yin *et al*. proved a more simple equivalent formation to the Bregman iterative regularization model in [6]. It is known from the numerical examples of [5] that the Bregman iterative regularization method can keep the horizontal and the vertical edges well and the bent edges badly. On the contrary, we see that Chambolle’s dual denoising method in [4] can keep the curve well and the horizontal and the vertical edges badly. Accordingly, in this paper, we give a comprehensive denoising method based on the dual denoising algorithm [4] and the Bregman iterative regularization method [5, 6]. In this paper, implicitly assumed, dual denoising just refers to Chambolle’s dual denoising algorithm or the gradient projection method.

Firstly, we choose a proper weight parameter *β* and modify the ROF functional to a modified form with a weighted taking-back-noise term:

$$b_{k + 1} = (g + b_{k}) + \beta\pi_{\lambda K}(g + b_{k}). $$

The weight parameter $\beta\in(0,1)$, maintains a balance between the Bregman iterative regularization method and the dual denoising method. The value of *β* varies according to the noise level and it is approximately inversely proportional to the noise level. Specially, when $\beta= 0$, we solve the ROF model by the gradient projection method for there is no information that is taken back to the model. As for $\beta = 1$, the model becomes the Bregman iterative regularization model. Secondly, we iteratively solve the modified ROF model until the end condition is met. When $0 < \beta< 1$, we solve the modified ROF model by Chambolle’s dual algorithm. The results of the numerical experiments demonstrate that the new method cannot only restore more straight edges than the dual denosing method but also restore more bent edges than the Bregman iterative regularization method.

The rest of this paper is organized as follows. In Section 2, we briefly review the dual denoising method and Bregman iteration denoising method. In Section 3, we propose our weighted gradient projection denoising method. Then, in Section 4, we apply our new method to 1-D curve, 2-D gray image and 3-D color image denoising examples, respectively, and present the numerical results. Finally, we give a conclusion.

## Preliminaries

### Dual denoising method

Noticing that

2.1 $$ \operatorname{TV}(u) = \sup_{v \in K}(u,v)_{X} $$

(1.2) can be rewritten as

2.2 $$ \min_{u}J(u) + \frac{1}{2\lambda} \Vert u - g \Vert _{2}^{2}. $$

The Euler equation for (2.2) is

2.3 $$ 0 \in u - g + \lambda\partial J(u), $$

which is equivalent to

$$(u - g) / \lambda\in\partial J(u) $$

and equivalent to

$$u \in\partial J^{ *} \bigl((g - u) / \lambda\bigr). $$

The above equation can be rewritten as

2.4 $$ 0 \in\frac{g - u}{\lambda} - \frac{g}{\lambda} + \frac{1}{\lambda} \partial J^{ *} \biggl(\frac{g - u}{\lambda} \biggr), $$

where $J^{ *} $ is the Legendre-Fenchel transform of *J* [10–12], defined by

2.5 $$ J^{ *} (v) = \sup_{u}(u,v)_{X} - J(u). $$

#### Proposition 2.1

2.6 $$ J^{ *} (v) = \chi\kappa= \left \{ \textstyle\begin{array}{l@{\quad}l} 0 &\textit{if }v \in\kappa, \\ + \infty& \textit{otherewise}. \end{array}\displaystyle \right . $$

Let $\omega= (u - g) / \lambda$, (2.4) is the Euler equation for the minimization problem

$$\min_{\omega} \frac{ \Vert \omega- (g / \lambda) \Vert _{2}^{2}}{2\lambda} + \frac{1}{\lambda} J^{ *} (\omega). $$

By Proposition 2.1, we get $\omega= \pi_{K}(g / \lambda)$. The solution of equation (2.2) can be simplified as

2.7 $$ u = g - \pi_{\lambda K}(g). $$

For computing *u*, we just need to compute the nonlinear projection $\pi_{\lambda K}(g)$, *i.e*. to solve the following problem:

2.8 $$ \min\bigl\{ \big\Vert \lambda \operatorname{div}p - g) \big\Vert _{2}^{2}: \forall p \in Y, \vert p_{i,j} \vert ^{2} - 1 \le0,i=1, \ldots, M, j=1, \ldots, N \bigr\} , $$

here, *M*, *N* represent the total number of pixels in each row and in each column.

Given $\lambda= \lambda_{0} > 0$, $0 < \tau< \frac{1}{8}$, $p^{0} = 0$, for any $n \ge0$, Chambolle’s gradient projection method for the denoising problem (2.7) is described as below.

$$\begin{aligned}& \textbf{for } n = 0, \ldots,L_{\mathrm{out}} \\& \quad \textbf{Initialization}\text{: } p = 0; \\& \quad\textbf{for } t = 0, \ldots,L_{\mathrm{in}} \\& \quad\quad p_{i,j}^{n + 1} = \frac{p_{i,j}^{n} + \tau(\nabla(\operatorname{div}p^{n} - g / \lambda_{t}))_{i,j}}{1 + \tau \vert (\nabla(\operatorname{div}p^{n} - g / \lambda_{t}))_{i,j} \vert }; \\& \quad\quad v_{n + 1} = \pi_{\lambda_{t}}(g) = \lambda_{t}\operatorname{div}p^{n + 1}; \\& \quad\quad f_{n + 1} = f_{n + 1}(\lambda_{t}) = \big\Vert \pi_{\lambda_{t}K}(g) \big\Vert = \Vert v_{n + 1} \Vert ; \\& \quad\textbf{end} \\& \quad\lambda_{t} = \frac{N\sigma}{f_{n + 1}}\lambda_{t}; \\& \textbf{end} \\& \lambda_{t + 1} = \lambda_{t}; \\& u = g - v_{n + 1} \quad\bigl(\textit{i.e. }u = g - \pi_{\lambda_{t + 1}K}(g)\bigr). \end{aligned}$$

Here $I_{\mathrm{out}}$, $I_{\mathrm{in}}$ denote the iterative numbers of the external iterations and the internal iterations time for *u*. $N ^{2}$ is the total number of pixels. *σ* is the noise standard deviation. For convenience, we set the inner loop times $L_{\mathrm{in}}$ and the outside loop time $I_{\mathrm{out}}$.

#### Lemma 2.1

([4])

*Let* $0 < \tau< \frac{1}{8}$ *and* $p^{0} = 0$, *then*, *for any* $\lambda_{0} > 0$, $\lambda \operatorname{div}p^{n}$ *converges to* $\pi_{\lambda_{ n} K}(g)$ *as* $n \to+ \infty$.

### Bregman iterative regularization denoising method

Osher *et al*. proposed a Bregman iterative regularization denoising method and proved the convergence [5]. A simple and equivalent iterative procedure to the BRI denoising model was given in [8], and the convergence of this simplified method was also analyzed. Here we consider the simplified BRI denoising model in [8]. The simplified BIR denoising model is as follows:

2.9 $$ u_{k + 1} = \mathop{\mathit{art}\min}_{u \in \operatorname{BV}(\Omega)}\bigl\{ \vert u \vert _{\mathrm{BV}} + \mu \Vert g + b_{k} - u \Vert _{L^{2}}^{2}\bigr\} , $$

where $\operatorname{BV}(\Omega)$ denotes the space of functions with bounded variation on Ω and $\vert \cdot \vert _{\mathrm{BV}}$ denotes the BV seminorm, formally given by

$$\vert u \vert _{\mathrm{BV}} = \int_{\Omega} \vert u \vert , $$

which is also referred to as the total variation (TV) of u, and update

2.10 $$ b_{k + 1} = b_{k} + g - u_{k + 1}, $$

where $b_{k}$ is the information taken back (we set $b_{0} = 0$), *g* is the degenerative image contaminated by additive white gaussian noise.

The Bregman iteration technique has the advantage of converging quickly when applied to certain types of objective functions and the advantage of keeping a fixed value of *λ* as denoisings [8].

#### Lemma 2.2

([8])

*Suppose that some iterate*, $u^{*}$, *satisfies* $Au^{*} = b$. *Then* $u^{*}$ *is a solution to the constrained problem* (2.9).

## A weighted denoising method

While the gradient projection and the BIR denoising methods are extremely efficient, they can either keep the straight edges or keep the bent curves well. From the denoised results of [5] and [4], we see that the bent parts of the curve do not get restored perfectly by the BIR method, while the straight edges are not be kept well by Chambolle’s dual denoising method. So we plan to combine these two methods to improve the restored efficiency of the noisy images. We found that the denoising effects were not very good if we just put these two methods together. This is because too much noise was taken back if the noise level is heavy. So we propose a weighted coefficient strategy to eliminate this phenomenon.

Firstly, we use the simplified Bregman iterative regularization model:

$$u_{k + 1} = \mathop{\mathit{art}\min}_{u \in \operatorname{BV}(\Omega)}\bigl\{ \vert u \vert _{\mathrm{BV}} + \mu \Vert g + b_{k} - u \Vert _{L^{2}}^{2}\bigr\} ; $$

for the sake of consistency, setting the parameter *μ* in the above functional equal to $\frac{1}{2\lambda} $ we consider the modified ROF model

3.1 $$ u_{k + 1} = \mathop{\mathit{art}\min}_{u \in \operatorname{BV}(\Omega)}\biggl\{ \vert u \vert _{\mathrm{BV}} + \frac{1}{2\lambda} \Vert g + b_{k} - u \Vert _{L^{2}}^{2}\biggr\} ; $$

if we apply Chambolle’s dual algorithm to each iteration of (3.1) by Chambolle’s dual algorithm, we have

$$u_{k + 1} = g + b_{k} - \pi_{\lambda K}(g + b_{k}); $$

and the update

$$b_{k + 1} = b_{k} + (b_{k} + g - u_{k + 1}). $$

By a simple derivation, we have

3.2 $$ b_{k + 1} = b_{k} + \pi_{\lambda K}(g + b_{k}), $$

where $b_{0} = 0$.

*Bregman-gradient projection method initialize*:

$$\begin{gathered} \textbf{Initialization}\text{: }u_{0} = g,\qquad b_{0} = 0,\qquad \lambda= \lambda_{0}\quad (\text{here, we choose a }\lambda_{0} > 0 ) \\\textbf{While }\Vert u_{k} - u_{k - 1} \Vert _{2} > \textit{tol} \quad(\textit{tol}\text{ is the tolerance}) \\\quad\textbf{for } k = 0 ,\ldots, K^{*}\text{:} \\ \quad\text{Using Chambolle's dual method to compute $u_{k + 1}$ in (3.1), we obtain} \\\quad u_{k + 1} = (g + b_{k}) - \pi_{\lambda K}(g + b_{k}) \\ \quad\textbf{end} \\ \quad\text{and using the new update (3.2):} \\\quad b_{k + 1} = b_{k} + \pi_{\lambda K}(g + b_{k}) \\ \textbf{end}\end{gathered}$$

For simplicity, we preset the outside recycling (*i.e*. the Bregman iteration) numbers and the internal recycling (*i.e*. the dual iteration) numbers. Usually we just need 1 or 2 steps outside recycling. It is easy to see that we just need to replace *g* in (2.7) by $g + b_{k}$. This mixed denoising method is mainly based on the Bregman iterative regularization denoising and Chambolle’s gradient projection denoising method, which ensures that each sub-problem has a closed-form solution. However, if we just put these two methods together, the denoising effects were not very good.

Secondly, we add a weight factor *β* before the taking-back-noise term of the updating iteration step, *i.e*.

3.3 $$ b_{k + 1} = b_{k} + \beta\pi_{\lambda K}(g + b_{k}). $$

Here, the weighted coefficient $\beta\in[0,1]$, $\beta\ge0$, is used to balance the amount of the noises taken back to the latest denoised result. The strategy is that the bigger the noise level, the smaller the *β* is. This is because too much noise was taken back if the noise level is heavy. Next, we will give the mixed denoising method of BIR denoising and the dual denoising method.

*Weighted Bregman-gradient projection method*

$$\begin{aligned}& \textbf{Initialization}\text{: }u_{0} = g,\qquad b_{0} = 0,\qquad \lambda= \lambda_{0}\quad (\text{here, we choose a }\lambda_{0} > 0 ) \\& \textbf{While } \Vert u_{k} - u_{k - 1} \Vert _{2} > \mathit{tol} \\& \quad\textbf{for } k = 0, \ldots, K^{*}\text{:} \\& \quad\text{Using Chambolle's dual method to compute $u_{k + 1}$ in (3.1), we obtain} \\& \quad u_{k + 1} = (g + b_{k}) - \pi_{\lambda K}(g + b_{k}) \\& \quad\textbf{end} \\& \quad\text{and using the new update (3.2):} \\& \quad b_{k + 1} = b_{k} + \beta\pi_{\lambda K}(g + b_{k}) \\& \textbf{end} \end{aligned}$$

Through experiments, we give the value of *β*. In the 1-D and 2-D cases we select $\beta= 0.4$ and $\beta= 0.1$, respectively. In 3-D cases, when $\sigma= 12$, we choose $\beta= 0.1$; and when $\sigma= 25$, we choose $\beta= 0.05$. All the PSNRs of our method in 1-D, 2-D and 3-D are bigger than those of the dual algorithms. The results presented in this paper extend and improve the related results of [5] and [4]. The details of the parameter *β* are showed in Table 1.

**Table 1:** **Values of the parameter** ***β***

**Dimension**	**1-D**	**2-D**	**3-D**	
			***σ*** ** = 12**	***σ*** ** = 25**
*β*	0.4	0.1	0.1	0.05

## Numerical experiments and discussions

In this section, we will examine the effectiveness of the weighted Bregman method on TV denoising. The new method was implemented in FORTRAN and MATLAB, and compiled on a Win7 platform.

Firstly, we test our method by denoising three kinds of images: the 1-D curves, the 2-D gray images and the 3-D color images.

Next, we will compare the peak signal-to-noise ratios (PSNRs) of the dual denoising method and our new denoising method. Under the same number of iterations, all the results show that the PSNRs of the new method are higher than those of the dual denoising method. Here, the PSNR is defined as follows: given a noise-free *m*-by-*n* image *I* and its noisy approximation *K*,

$$\mathit{PSNR} = 10 \cdot\log_{10}\biggl(\frac{\mathit{MAX}_{I}^{2}}{\mathit{MSE}}\biggr), $$

where the mean squared error (MSE) is defined as

$$\mathit{MSE} = \frac{1}{mn}\sum_{i = 0}^{m - 1} \sum _{j = 0}^{n - 1} \bigl[I(i,j) - K(i,j) \bigr]^{2}, $$

where $\mathit{\mathit{MAX}} _{I}$ denotes the maximum possible pixel value of the image. When the pixels are represented using 8 bits per sample, $\mathit{\mathit{MAX}} _{I}$ is 255.

### Example 1

**Figure 1:**
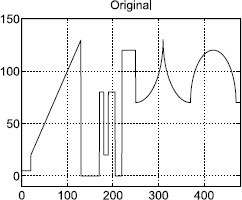
**1-D original image.**

**Figure 2:**
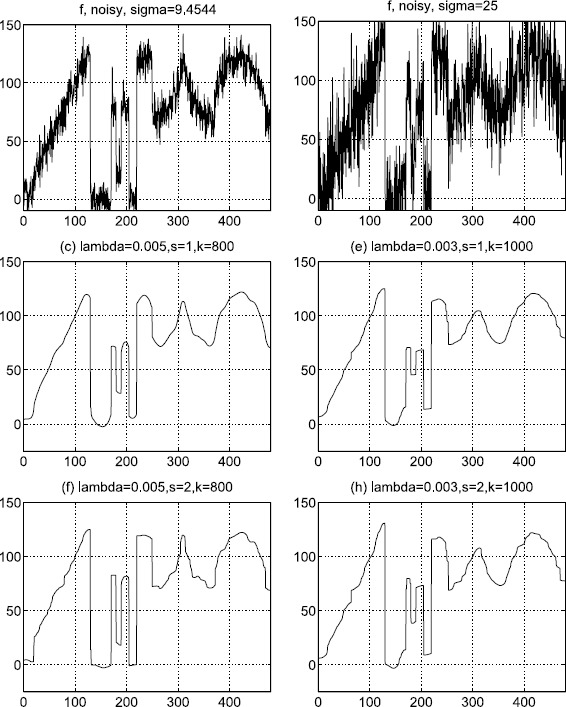
**1-D denoising results.**

In our first test, an 1-D signal *f* (Figure 1) is used. We add Gaussian white noises of standard deviation 9.4544 and 25 on *f*. The corresponding degraded images are displayed in the top row of Figure 2 from left to right, respectively. All the 1-D restored results of the dual method and the proposed method are shown in Figure 2 also. The restored results for the dual denoising method are shown in the middle row of Figure 2, and the PSNRs are 36.3555 dB (left) and 31.1127 dB (right). The denoised images for the proposed method are shown in the bottom row of Figure 2. The PSNRs are 40.1584 dB (left) and 32.8930 dB (right), respectively. In comparison, it shows that the restored output images of the proposed method are very satisfactory. In fact, under the same conditions, the proposed method can reconstruct more details of the straight edges and the cusps than the dual method. Here, ‘$s-1$’ is the taking-back-noises iteration time and ‘*k*’ refers to the dual iteration number. The dual iterations stop when the infinite module of the denoised results of the *k*th step and $(k + 1)$th step is less than 0.01.

### Example 2

**Figure 3:**
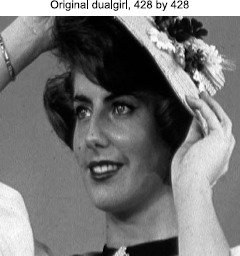
**2-D original image.**

**Figure 4:**
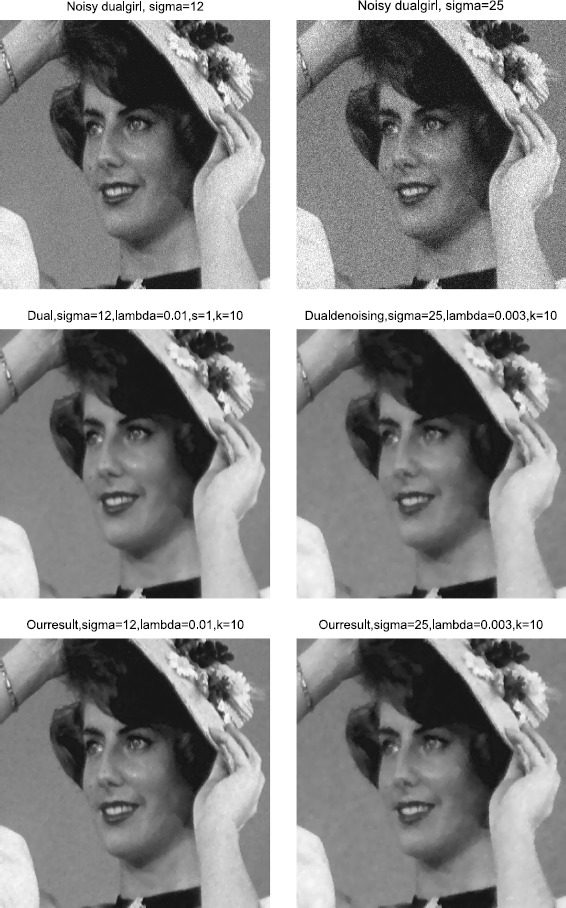
**2-D denoising results.**

In the second example, we further test the effectiveness of the proposed method by denoising the gray images. The clean 2-D gray image of Figure 3 is contaminated by gaussian white noise with standard deviation 12 and 25, and the noisy images are shown in the first row of Figure 4 from left to right. A comparison of the denoising results for the dual method and the proposed method is provided in the last two rows of Figure 4. In the middle row of Figure 4, the PSNRs for the dual method are 33.9067 dB (left) and 31.1647 dB (right). The bottom images are the restored images using the proposed method, with PSNRs 34.3631 dB (left) and 31.2651 dB (right). The denoising parameter of *A* from left to right is 0.01 and 0.003. Also here, the symbol *k* refers to the dual iteration number. All the taking-back-noises iteration numbers are one. Comparison results show again that the contours and the details such as the girl’s hair, mole, nose and teeth are recovered more clearly by our new method than the dual method.

### Example 3

**Figure 5:**
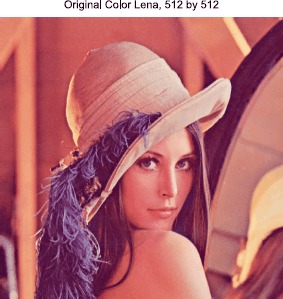
**3-D original image.**

**Figure 6:**
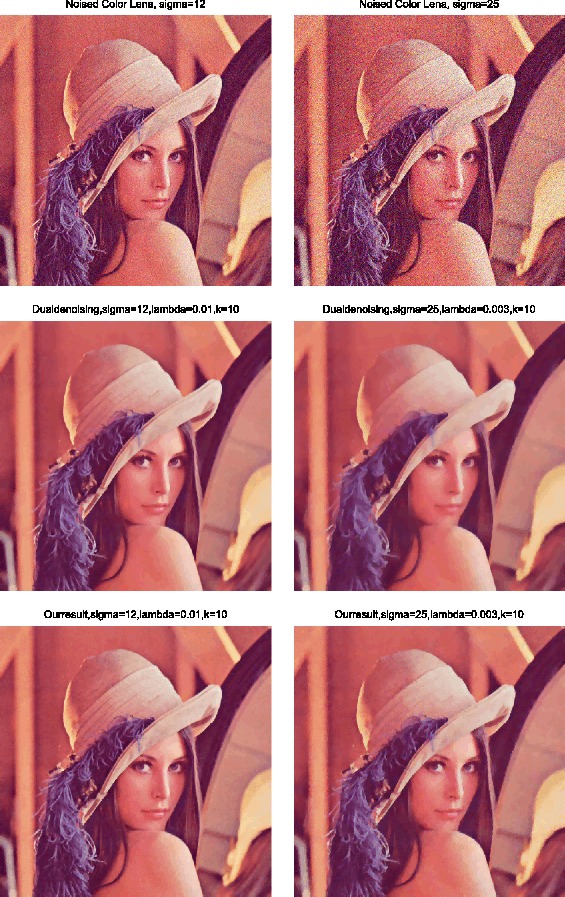
**3-D Denoising results.**

In this experiment, we come to deal with 3-D color image. The original RGB ‘Lena’ image in Figure 5 is distorted by gaussian white noises with standard deviation of 12 and 25, respectively. The noisy images are shown in the Figure 6 at the top left and the top right. In Figure 6, the second row, the denoised results for the dual method are shown, the PSNR of the left one is 32.1209 dB and the right one is 28.7921 dB. In the third row, the restoration results by the proposed method are displayed, with the PSNRs 32.7622 dB (left) and 29.6473 dB (right) severally. The denoising parameter of *λ* from left to right is 0.01 and 0.003, the taking-back-noises iteration number equals one and the dual iteration number *k* is ten. It is clear that, no matter the noise level is light or heavy, the restored results by our method are much better than those by the dual method, especially in the contours and details, such as the hair and the hat.

In Table 2, we present the PSNRs of the dual algorithm and our new method. Here *σ* is the noise standard deviation.

**Table 2:** **PSNR results of Chambolle’s dual method and our new method**

**Dimension**	***σ***	**Dual (dB)**	**Ours (dB)**
**1-D**	*σ* = 9.4544	36.3555	40.1584
	*σ* = 25	31.1127	32.8930
**2-D**	*σ* = 12	33.9067	34.3631
	*σ* = 25	31.1647	31.2651
**3-D**	*σ* = 12	32.1209	32.7622
	*σ* = 25	28.7921	29.6473

## Conclusions

In this paper, we proposed a weighted Bregman-gradient projection denoising method. Several kinds of images are denoised by the new method. Numerical results indicate that the new method is more accurate than the dual denoising method and Bregman iteration regularized method.

## References

[1] Rudin LI, Osher S, Fatemi E (1992). Nonlinear total variation based noise removal algorithms. Phys. D, Nonlinear Phenom..

[2] Aubert G, Kornprobst P (2002). Mathematical Problems in Image Processing: Partial Differential Equations and the Calculus of Variations.

[3] Chan T, Shen J (2005). Image Processing and Analysis: Variational, PDE, Wavelet, and Stochastic Methods.

[4] Chambolle A (2004). An algorithm for total variation minimization and applications. J. Math. Imaging Vis..

[5] Osher S, Burger M, Goldfarb D, Xu J, Yin W (2011). An iterated regularization method for total variation-based image restoration. IEEE International Conference on Imaging Systems and Techniques.

[6] Yin W, Osher S, Goldfarb D, Darbon J (2008). Bregman iterative algorithms for *l*1-minimization with applications to compressed sensing. SIAM J. Imaging Sci..

[7] Chang Q, Chern IL (2003). Acceleration methods for total variation-based image denoising. SIAM J. Sci. Comput..

[8] Goldstein T, Osher S (2009). The split Bregman method for L1 regularized problems. SIAM J. Imaging Sci..

[9] Jia R, Zhao H (2010). A fast algorithm for the total variation model of image denoising. Adv. Comput. Math..

[10] Rockafellar RT (1972). Convex Analysis.

[11] Boyd S, Vandenberghe L (2004). Convex Optimization.

[12] Ekeland I, Temam R (1999). Convex Analysis and Variational Problems.

